# The Florence Crittenton Home

**Published:** 1893-11

**Authors:** 


### THE FLORENCE CRITTENTON HOME.

Through the liberality of one of the advertising patrons of this journal, a very excellent and worthy institution has lately been added to the public charities of Atlanta. The Florence Crittenton Home for fallen women was formally opened and presented to the city October 17. In it will be received such young women as have become pregnant out of wedlock, with the view of reclaiming them from a subsequent life of shame. The institution will be conducted after the manner of those established by Mr. Crittenton in other cities. They have been found in other places to supply a great necessity in rescuing hundreds of women whose lives would have been lost to virtue and honor. The Atlanta home has already sheltered thirty-one girls, and has had under its care nineteen children. The home is under the very best management and is accomplishing a very excellent work.

